# A Review of Wearable Back-Support Exoskeletons for Preventing Work-Related Musculoskeletal Disorders

**DOI:** 10.3390/biomimetics10050337

**Published:** 2025-05-20

**Authors:** Yanping Qu, Xupeng Wang, Xinyao Tang, Xiaoyi Liu, Yuyang Hao, Xinyi Zhang, Hongyan Liu, Xinran Cheng

**Affiliations:** 1 School of Mechanical and Precision Instrument Engineering, Xi’an University of Technology, Xi’an 710048, China; quyanping@xpu.edu.cn; 2 Industrial Design Department, Xi’an University of Technology, Xi’an 710048, China; 2220621035@stu.xaut.edu.cn (X.L.); 2220621011@stu.xaut.edu.cn (Y.H.); 2220621005@stu.xaut.edu.cn (X.Z.); 2230620040@stu.xaut.edu.cn (H.L.); 2230620042@stu.xaut.edu.cn (X.C.)

**Keywords:** manual material handling, work-related musculoskeletal disorders, low back pain, wearable exoskeleton, lumbar spine assistance

## Abstract

Long-term manual material handling (MMH) work leads to the trend of the younger onset of work-related musculoskeletal disorders (WMSDs), with low back pain (LBP) being the most common, which causes great trouble for both society and patients. To effectively prevent LBP and provide support for workers engaged in MMH work, wearable lumbar assistive exoskeletons have played a key role in industrial scenarios. This paper divides wearable lumbar assistive exoskeletons into powered, unpowered, and quasi-passive types, systematically reviews the research status of each type of exoskeleton, and compares and discusses the key factors such as driving mode, mechanical structure, control strategy, performance evaluation, and human–machine interaction. It is found that many studies focus on the assistive performance, human–machine coupling coordination, and adaptability of wearable lumbar assistive exoskeletons. At the same time, the analysis results show that there are many types of performance evaluation indicators, but a unified and standardized evaluation method and system are still lacking. This paper analyzes current research findings, identifies existing issues, and provides recommendations for future research. This study provides a theoretical basis and design ideas for the development of wearable lumbar assistive exoskeleton systems.

## 1. Introduction

With the rapid advancement of technology, robots and automated equipment have become widely used in modern factories to replace manual operations. However, there are still many roles that require manual material handling (MMH) of heavy objects, such as logistics freight loading and unloading, airport cargo transit, assembly line heavy lifting, and construction material handling [1]. Prolonged manual handling can lead to various work-related musculoskeletal disorders (WMSDs), mainly affecting the neck, shoulders, and lower back, representing one of the most severe occupational health issues [2]. Thus, improving working conditions for these roles and reducing physical strain is critical.

According to a global burden of disease analysis, approximately 1.71 billion people worldwide suffer from musculoskeletal disorders [3]. Among them, high-income countries account for the largest number of affected individuals, with 441 million cases, followed by the Western Pacific region (427 million) and the Southeast Asian region (369 million), according to the World Health Organization. Musculoskeletal disorders are also the main cause of disability globally, with approximately 149 million disabilities attributed to them, representing 17% of the global total. Among these, low back pain (LBP) is the most common form of WMSD caused by long-term heavy lifting and manual handling [4]. Most people experience some form of LBP during their lifetime, with the incidence peaking around 30 years of age [5]. Studies have shown that LBP prevalence may range from 4% to 69% [6]. Extended periods of manual heavy lifting can increase the risk of LBP among workers in various industrial sectors, leading to significant impacts on workers’ quality of life and considerable economic and productivity losses. So, industrial exoskeletons have been developed to reduce the physical demands of workers in the workplace and mitigate ergonomic issues associated with WMSDs [7].

**Figure 1:**
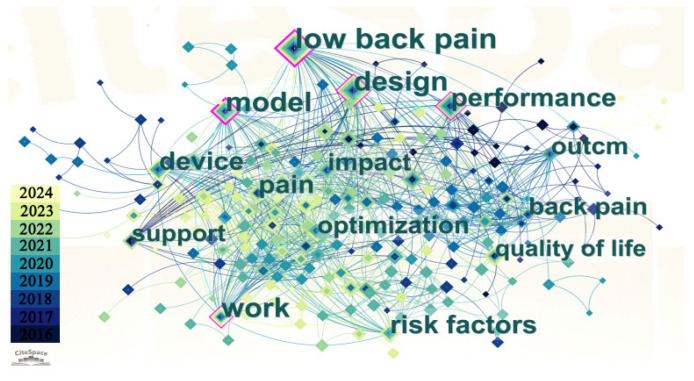
Keyword clustering map.

**Figure 2:**
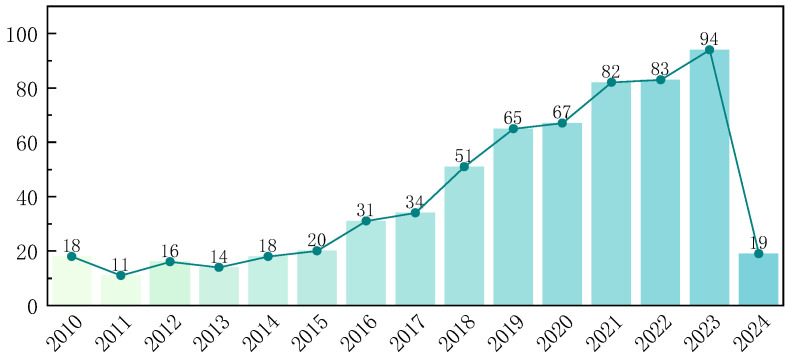
Statistics on the number of publications from 2010 to 2024.

This paper focuses on a systematic review of studies related to wearable lumbar support exoskeletons, which were published from 2010 to 2024. Using keywords such as “Industrial exoskeletons” “Assistive devices” “Lumbar exoskeletons” “Wearable robots”, and “Back support devices”, a search was conducted in the Web of Science (WOS) Core Collection, as illustrated in Figure 1. By using Citespace V6.3 software to analyze clustering in the titles and abstracts of 623 research articles, this research obtained a network evaluation index of Q = 0.564, indicating a good clustering effect. The primary research surrounding wearable lumbar support exoskeletons focused on low back pain, design, performance, modeling, and support, and so on. The volume of related research publications has steadily increased, reaching a peak in the past three years, as depicted in Figure 2.

This study reviews the research progress of wearable lumbar assisted exoskeleton in preventing musculoskeletal diseases related to handling work. The research status of electric, unpowered, and quasi-passive lumbar exoskeletons is systematically analyzed, and the key factors such as driving mode, mechanical structure, control strategy, performance evaluation, and human–computer interaction are compared and discussed. By summarizing the current research situation at home and abroad, analyzing the existing problems, and looking forward to the future development direction, it provides theoretical reference and design ideas for the development of wearable lumbar auxiliary exoskeleton system.

## 2. Methods

### 2.1. Search Strategy

This study conducts a systematic review of wearable lumbar support exoskeletons following the Preferred Reporting Items for Systematic Reviews and Meta-Analyses (PRISMA) guidelines. Articles were retrieved from three online databases—Web of Science, Scopus, and ProQuest—for the period from 1 January 2010 to 1 March 2024. The search utilized keywords including “industrial exoskeletons” “assistive devices” “lumbar exoskeletons” “wearable robots”, and “back support devices”. Additional searches were manually conducted to identify relevant references and abstracts from recent conferences, including through Google Scholar, to ensure a comprehensive collection of related research.

### 2.2. Study Eligibility Selection

The inclusion criteria for this study were defined as follows: (1) designs of lumbar support exoskeletons, (2) back support devices in industrial settings, and (3) performance evaluations of lumbar support exoskeletons. The exclusion criteria comprised the following: (1) non-human studies, (2) exoskeletons applied to body parts other than the lumbar region, (3) studies based solely on simulation results, (4) orthotic devices, (5) studies relying solely on subjective evaluation, and (6) review articles.

### 2.3. Study Screening

**Figure 3:**
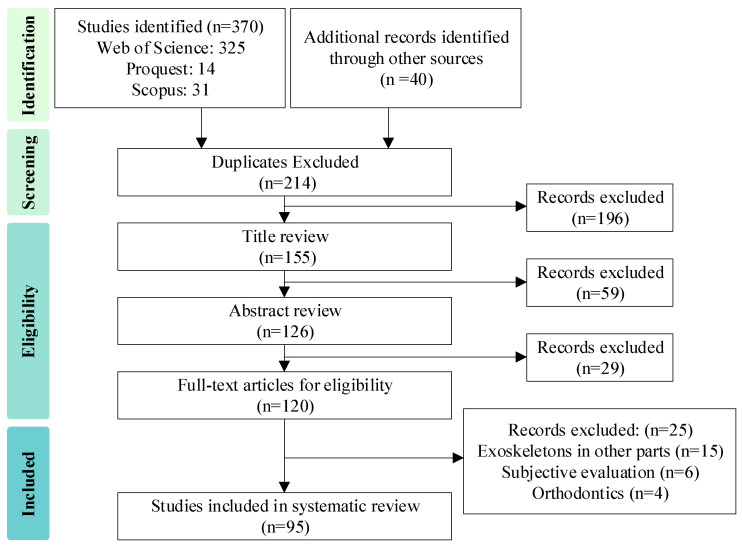
The PRISMA flow diagram of the study search and screening process.

The initial screening process involved reviewing the titles to remove unrelated studies. In the second screening, abstracts were read to further filter out studies based on the exclusion criteria. From this process, 214 articles were selected for full-text review. The scope was then refined to focus exclusively on lumbar exoskeletons, and these studies were analyzed in depth with an emphasis on their mechanical design, assistance performance, and the methods used for measurement and validation. Specifically, n = 95 articles were analyzed in detail for this review. The data collection and selection process are presented in the PRISMA flowchart shown in Figure 3.

## 3. Results

**Figure 4:**
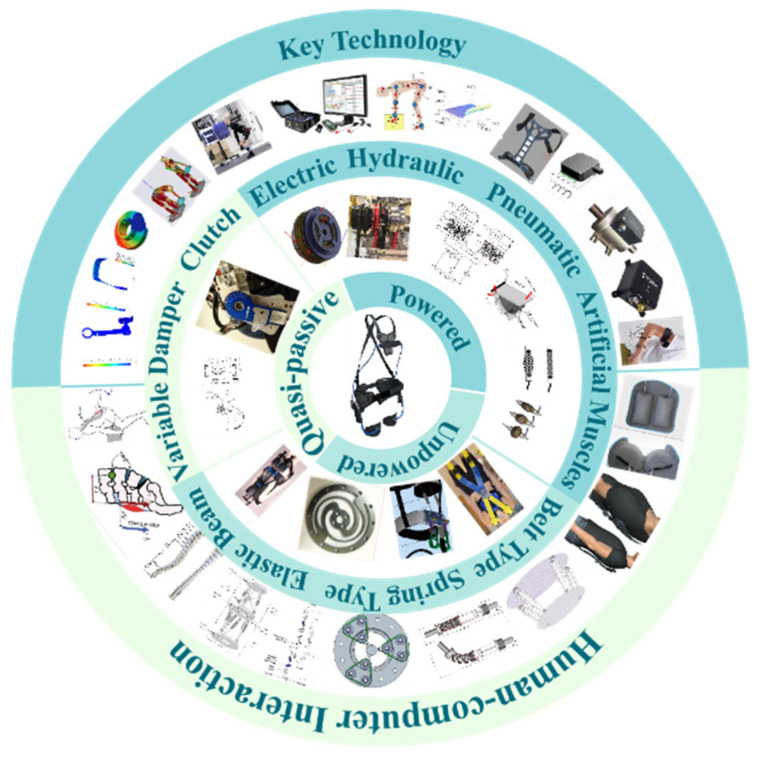
The research topic.

Lumbar support exoskeleton technology is becoming increasingly popular in physically demanding jobs, such as logistics sorting, agricultural labor, and medical care [8]. Its overarching goal is to reduce the muscle load on the lower spine and joint torques, as well as shearing and compressive forces. As shown in Figure 4, based on their power sources, lumbar support exoskeletons can be categorized into three types: powered, unpowered, and quasi-passive. Powered exoskeletons use components that can generate torque (such as motor drives), while unpowered exoskeletons primarily rely on passive elastic structures to store energy. Quasi-passive exoskeletons are an intermediate form, using passive elastic structures to store and release energy, with a lower demand for power compared to powered types and without direct influence on gait.

All types of exoskeletons aim to provide wearers with additional trunk extension torque to reduce the stress and fatigue on the lumbar region during manual handling tasks. This paper aims to conduct a comprehensive analysis of the research status of lumbar support exoskeletons, focusing on key technologies such as actuation methods, mechanical structures, control strategies, and human–machine interactions for powered, unpowered, and quasi-passive lumbar support exoskeletons. By summarizing research in these areas, providing valuable insights for future studies on lumbar support exoskeletons.

### 3.1. Biomechanical Characterization Analysis

**Figure 5:**
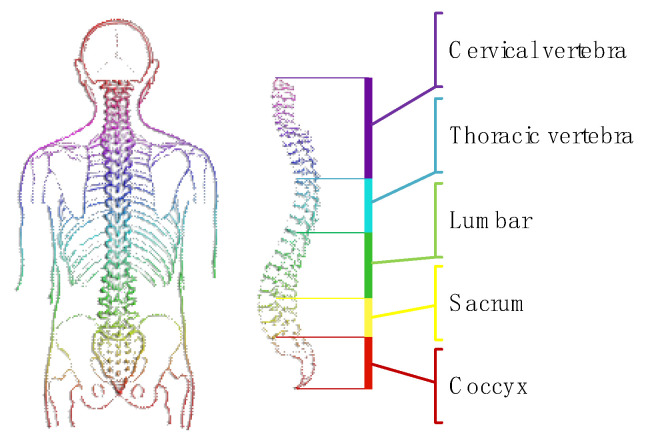
A diagram of the human spine.

In daily life, material handling movements are highly cyclical. Lifting tasks typically involve bending, squatting, grasping, rising, and walking [9]. During these processes, the primary joints in motion are the lumbar spine and hip joints, as illustrated in Figure 5. The spine is composed of 33 vertebrae, with the spinal cavity, skull, and the joints between individual vertebrae allowing the body to move in multiple planes while maintaining structural integrity. Spinal movement is achieved through the antagonistic action of joints, ligaments, and multiple groups of muscles, with intervertebral disks providing connectivity and shock absorption.

### 3.2. Joint Range of Motion

**Figure 6:**
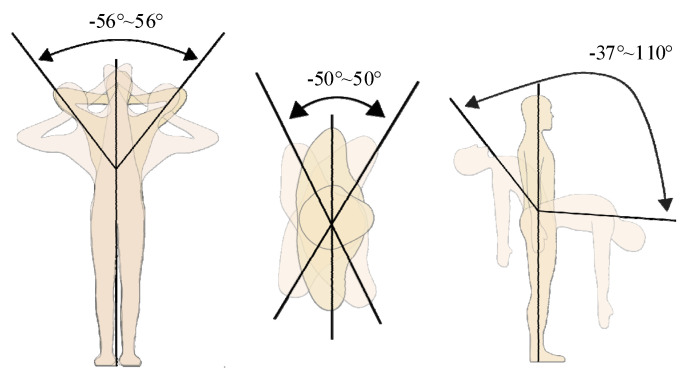
Waist motion diagram of human body.

The maximum range of motion for major joints during human handling tasks refers to the normal range of motion and is detailed in Table 1 [10]. The human body can be aligned using three mutually perpendicular planes: the sagittal plane, the frontal plane, and the transverse plane. In typical lifting and walking activities, most movement occurs in the sagittal plane, with minimal motion in the frontal and transverse planes, as depicted in Figure 6, which shows lumbar motion. Therefore, when designing lumbar support exoskeletons, the primary focus is on flexion and extension of key joints, and the limits of movement in these freedom degrees should not exceed the motion range joints. However, when analyzing actual material handling movements, two essential factors need to be considered comprehensively.

According to the measurement of lumbar motion of the human body, as shown in the figure above, lateral flexion is −56°~56°, rotation is −50°~50°, forward flexion is 110° and extension is 37°.

### 3.3. Biomechanical Analysis Based on Handling Movements

Biomechanical parameters are generally categorized into four types: spatiotemporal (time and distance), kinematic (joint displacement, angles, velocity, acceleration, etc.), dynamic (joint forces, torques, ground reaction forces), and biological (muscle force and electromyographic signal parameters). The division of motion phases is closely related to the changes in joint force, torque, and angle [11]. Panero E et al. [12] defined three motion phases for the material handling process.

**Figure 7:**
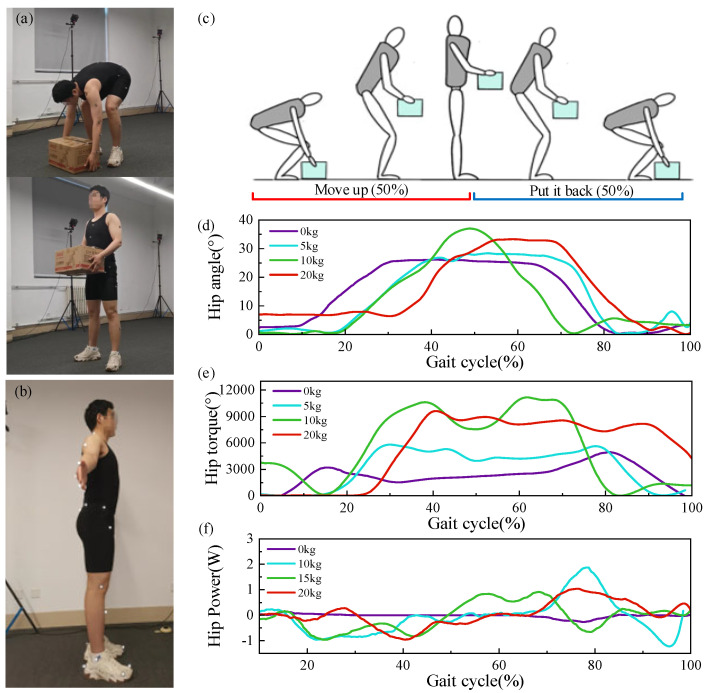
The experiment and results of the material handling process. (**a**) The experimental motion setting (**b**) with the marker points for the dynamic capture experiment (**c**) with a total handling process defined. (**d**) Hip angle change during handling under different loads. (**e**) Hip torque change during handling under different loads. (**f**) Hip power change during handling under different loads.

The lowering phase, where the upper body bends to reach the object being handled, accounts for 30% of the entire handling cycle. The lifting phase, where the body remains bent and the hands grasp the heavy object in preparation for lifting, accounts for 40% of the handling cycle. The rising phase, where the lower back muscles provide extension force as the upper body extends and stands upright, accounts for 40% of the handling cycle. Using the Nokov motion capture system, motion data during the lifting process were obtained (Figure 7a), and Figure 7b shows the marker points used in the experiment. It was observed that changes in joint angles, joint torques, and joint powers were cyclical, and joint angle curves under different loads exhibited similar shapes with varying periodicity (Figure 7d). In the lifting experiments, a full cycle was defined as lifting an object from the ground and then placing it back on the ground (Figure 7c), with Figure 7e,f illustrating the changes in hip joint angles, torques, and power during the handling cycle under different loads.

**Figure 8:**
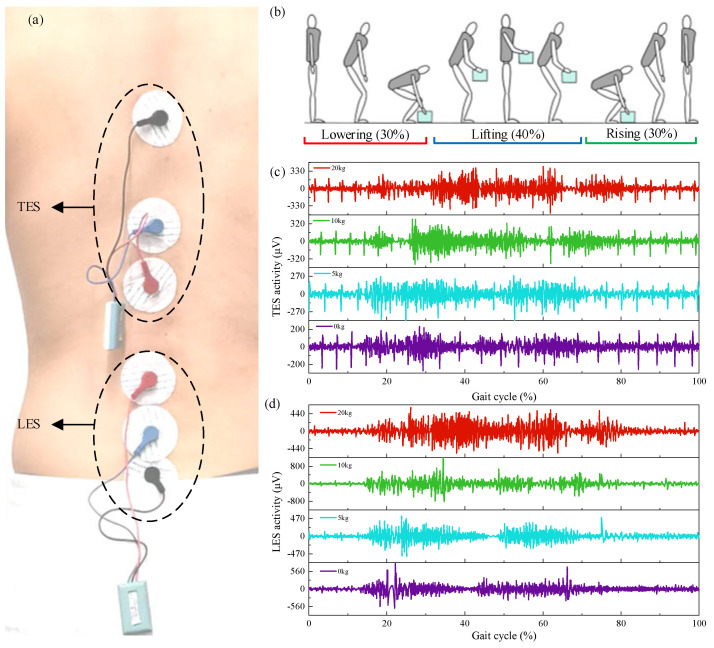
Transport EMG experiment. (**a**) Experimental electrode attachment. (**b**) Handling process (**c**) TES activation during handling under different loads. (**d**) LES activation during handling under different loads.

In the electromyography experiment, two muscles on the back, the thoracic erector spinae, and the lumbar erector spinae were tested (Figure 8a). The motion cycle was defined as bending down from a standing position to lift an object, then returning to the standing position, followed by placing the object back on the ground and returning to an upright stance (Figure 8b). Figure 8c,d show the activation patterns of the thoracic erector spinae (TES) and lumbar erector spinae (LES) during the handling cycle under varying loads.

### 3.4. Powered Lumbar Spine Assist Exoskeleton

Powered lumbar support exoskeletons rely on active drive systems to recognize human intent through sensors and other devices, providing assistive force to help wearers save energy during physical tasks. The power sources for these exoskeletons typically include electric motors, pneumatic systems, and hydraulic systems. Depending on the type of actuator used, the transmission mechanisms can vary in design and functionality. Based on the structural characteristics, lumbar support exoskeletons can be classified into rigid exoskeletons and flexible exoskeletons.

#### 3.4.1. Rigid Lumbar Spine Assist Exoskeleton

**Figure 9:**
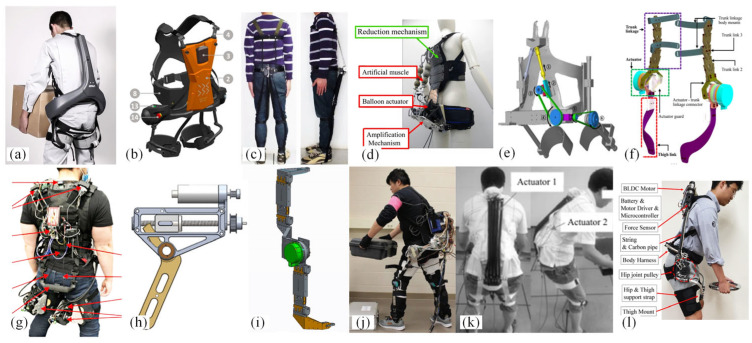
Powered lumbar spine assist exoskeleton. (**a**) Model Y [13]. (**b**) Cray X [14]. (**c**) WSAD [15]. (**d**) AB-Wear II [16]. (**e**) Back assistance exoskeleton [17]. (**f**) HipExo [18]. (**g**) ABX [19]. (**h**) APEx [20]. (**i**) Robo-Mate [21]. (**j**) Lower-back exoskeleton [22]. (**k**) Lower-back exoskeleton [23]. (**l**) Hip exoskeleton [24].

In 2018, the Japanese company Atoun developed the Model Y [13] (Figure 9a), based on their earlier Model A. The Model Y features an inverted “Y” design to minimize contact with the body while providing up to 10 kg of assistance. In the same year, the German company Bionic Systems, in collaboration with Fiat, developed the Cray X [14] (Figure 9b), a powered lumbar support exoskeleton. The Cray X utilizes motors and harmonic reducers for its operation and weighs 9 kg [14]. Both exoskeletons employ a combination of motors and harmonic gear transmissions, resulting in high costs and significant weight.

To address the issue of the substantial weight and inconvenience of prolonged use associated with motor-driven exoskeletons, Luo et al. [15] designed a wearable stooping-assist device (WASD) (Figure 9c). This model utilizes servo motors and tension bands to reduce weight, but it can compress muscles and provide limited assistance. Inose et al. [16] developed the waist assist suit called “AB-Wear”. AB-Wear II (Figure 9d) uses pneumatic artificial muscles and balloon actuators to control lumbar torque, weighing 2.9 kg.

Shin et al. [17] proposed a back exoskeleton powered by pneumatic artificial muscles (Figure 9e), which employs a gear and belt system to convert linear motion into rotational motion, thereby reducing mechanical energy loss. Perera et al. [18] developed a hip joint exoskeleton robot (the HipExo) (Figure 9f), designed for bending and lifting activities. This exoskeleton uses a hybrid actuator that combines a motor, a transmission, and a spring system to store and release energy, assisting with lifting tasks.

Due to the more severe lumbar injuries caused by asymmetric repetitive lifting, many devices have not addressed this issue. Li et al. [19] proposed the Asymmetric Back Exosuit (ABX, Figure 9g), which features a unique design and active cable drive. This allows for free movement and reduces the activation of the lumbar erector spinae muscles by 37.8% and 16.0% during symmetric and asymmetric lifting tasks, respectively.

To address the lifting and squatting actions during the handling process, Martin et al. [20] designed the Aerial Porter Exoskeleton (APEx, Figure 9h), which provides assistance through a combination of a hip-powered exoskeleton and lumbar support, thereby increasing hip torque.

#### 3.4.2. Flexible Lumbar Spine Assist Exoskeleton

In traditional industrial robot systems, rigid actuators are commonly used in scenarios requiring high precision, stability, and high torque bandwidth. However, their rigid transmission mechanism restricts the natural movements of the wearer, impacting the comfort of human–machine interaction. This limitation has led to a research focus on flexible exoskeletons.

To address the issues associated with rigid actuators, flexible actuators that allow deviation from preset positions have been developed, with series elastic actuators (SEAs) being the most prevalent. Robo-Mate [21] (Figure 9i) uses elastic actuators to provide lumbar support and motor assistance to offer additional torque, resulting in a relative reduction of about 28% in maximum voluntary contraction (MVC). Zhang et al. [22] (Figure 9j) designed a lower-back exoskeleton prototype where each drive unit includes a compact series elastic actuator, with the entire exoskeleton weighing 11.2 kg. However, exoskeletons using SEAs are generally bulky, so designing more compact and lightweight SEAs remains a challenge.

Flexible exoskeletons typically utilize flexible transmission mechanisms, such as pneumatic or cable-driven devices. Takuya et al. [25] developed a lower-back support exoskeleton, “Muscle Suit”, which uses rotary mechanisms and McKibben artificial muscles to provide assistive force to manual workers, significantly reducing the activity of the thoracic and lumbar erector spinae muscles. Xiangpan Li et al. [23] designed a wearable lower-back exoskeleton based on flexible pneumatic actuators (Figure 9k). Compared to McKibben pneumatic artificial muscles, the elongation-type pneumatic actuators are lighter, and the electromyography (EMG) signals decrease by about 29% during lifting and returning to an upright position.

Pneumatic-driven exoskeletons conform better to the human body curve compared to rigid structures but generally require external air sources and offer limited assistive performance. Flexible exoskeletons based on cable-driven mechanisms appear to hold more advantages. Seong et al. [24] proposed a design method for a hip joint exoskeleton based on a twisted string actuator (TSA), systematically designing a humanoid assistive mechanism (Figure 9l).

In summary, powered lumbar support exoskeleton robots primarily face issues such as motor-driven systems being complex and heavy. The lack of mechanisms conform to the lumbar region may lead to slippage and reduced contact area, decreasing comfort. Assistive devices using pneumatic artificial muscles provide better conformity than other drive methods but offer less assistive performance.

### 3.5. Non-Powered Lumbar Spine Assist Exoskeleton

#### 3.5.1. Belt Type

**Figure 10:**
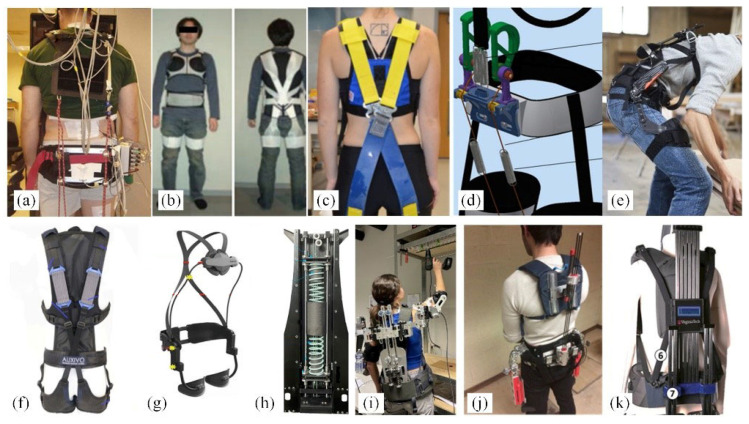
Non-powered lumbar spine assisted exoskeleton robot (**a**) PLAD [26]. (**b**) Smart Suit Lite [27]. (**c**) Lamers et al. [28]. (**d**) Passive spinal exoskeleton [29]. (**e**) Back X [30]. (**f**) LiftSuit2.0 [31]. (**g**) Leavo [32]. (**h**) Ding et al. [33]. (**i**) Upper-body exoskeleton [34]. (**j**) Spexor [35]. (**k**) VT-LOWE [36].

Frost et al. [26] developed the PLAD (personal lift assistive device, Figure 10a), designed to support back muscles during repetitive lifting tasks. This system uses elastic bands to mimic human muscles, transferring part of the force and torque to the shoulders, pelvic girdle, and knees, thereby reducing the load on the vertical spinal muscles. Imamura et al. [27] designed the Smart Suit Lite (Figure 10b), which utilizes the relationship between targeted movements and muscle forces to arrange and optimize the performance of the elastic bands. Lamers et al. [28] designed a prototype garment (Figure 10c) that works in parallel with the lumbar extensor muscles. By incorporating elastic bands, it generates forces parallel to the muscles and ligaments, supporting the lumbar extension torque.

The exoskeletons above use only elastic bands as elastic elements, which cannot be adjusted for different body sizes. In future research, elastic bands can be used as auxiliary components to increase assistive force, potentially achieving better results compared to using them as the sole assistive element.

#### 3.5.2. Spring Type

Using springs as assistive elements is one of the most widely adopted methods in unpowered lumbar support exoskeletons. Zhang et al. [29] designed a passive spinal exoskeleton (Figure 10d), which employs extension springs to implement a “push–pull” assist strategy at the thoracic and lumbar regions. Kazerooni et al. [30] developed the Back X (Figure 10e), utilizing gas springs to generate force, but it only provides assistance when the wearer bends. LiftSuit 2.0 [31] (Figure 10f) uses two textile springs parallel to the back muscles to provide support, reducing the activity of major back and gluteal muscles when leaning forward. The Leavo exoskeleton [32] (Figure 10g) combines gas springs and elastic tubes for assistance; however, these exoskeletons only offer support during bending and rising movements, which may interfere with the legs during walking. To address this issue, Ding [33] and Bettina et al. [34] designed a new type of passive exoskeleton (an upper-body exoskeleton) (Figure 10h,i), utilizing a spring-cable-differential mechanism to drive both hip joints, which provides additional user convenience.

#### 3.5.3. Elastic Beam Type

The passive back support exoskeleton Spexor proposed by Matthias et al. [35] (Figure 10j) provides up to 25 Nm of lower-back support torque [37]. Its trunk and hip horizontal misalignment compensation mechanism minimizes relative movement between the exoskeleton and the user. Virginia Tech collaborated with Lowe’s Inc. to develop the passive wearable exoskeleton VT-LOWE [36] (Figure 10k), which uses carbon fiber as an energy storage material. This exoskeleton can reduce peak and average activity of back muscles by approximately 31.5% and 29.3%, respectively [38].

In summary, unpowered lumbar support exoskeleton robots primarily utilize springs to achieve passive assistance, but they face several common issues, as follows.

(1) Limited assistance efficiency. The assistance provided by springs is difficult to control and depends solely on the bending angle, thus failing to offer sufficient assistance when lifting heavy objects.
(2) Load transfer. Although they reduce the burden on the lower back, the load is transferred to the lower limbs, potentially affecting other muscles.
(3) Comfort issues. Design elements such as constraints and limiting air cushions in exoskeletons may restrict freedom of movement and even cause discomfort in other muscles.

### 3.6. Quasi-Passive Lumbar Spine Assist Exoskeleton

Lumbar support exoskeletons typically employ structures with a single flexion–extension degree of freedom or a combination of primary and secondary passive elements to enhance system flexibility. However, the contradiction between structure and human body matching leads to increased control complexity and energy consumption [36]. Therefore, novel quasi-passive designs have emerged in recent years [39].

**Figure 11:**
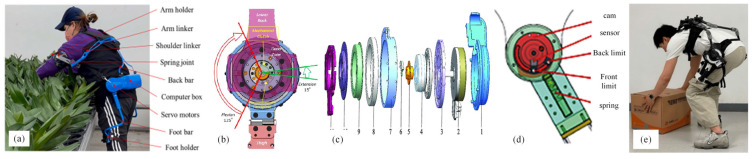
Quasi-passive lumbar power-assisted exoskeleton robot. (**a**) HExo [41]. (**b**) Powered lumbar exoskeleton [42]. (**c**) SIAT-WEXv1 [43]. (**d**) Quasi-passive energy storage lower-limb exoskeleton [44]. (**e**) Powered lumbar exoskeleton [45].

Quasi-passive lumbar support exoskeletons primarily utilize passive viscoelastic elements (such as springs and dampers) to provide support, while small actuators change the support level or disengage from passive elements [40]. A hybrid exoskeleton (HExo) designed by Le et al. combines a passive upper limb exoskeleton with an active lower-back exoskeleton, driven by battery-powered servo motors [41] (Figure 11a). Yong et al. employ a mechanical clutch to achieve the combination of primary and secondary passive elements, reducing energy consumption and improving usage time [42] (Figure 11b). The waist exoskeleton of Shenzhen Institutes of Advanced Technology (SIAT-WEXv1) developed by Yong et al. only employs motors when assistance is required, reducing energy consumption [43] (Figure 11c). Ma Jianfeng et al. proposed a quasi-passive energy-storing lower-limb exoskeleton, which reasonably allocates the triggering position and timing of energy release [44] (Figure 11d). In addition, Song et al. use a compact variable gravity compensation (CVGC-II) mechanism, which allows the AD exo-Back Support (AeBS) quasi-passive exoskeleton to provide various orders of auxiliary torque to the human body without limiting the range of motion [45] (Figure 11e).

## 4. Discussion

**Figure 12:**
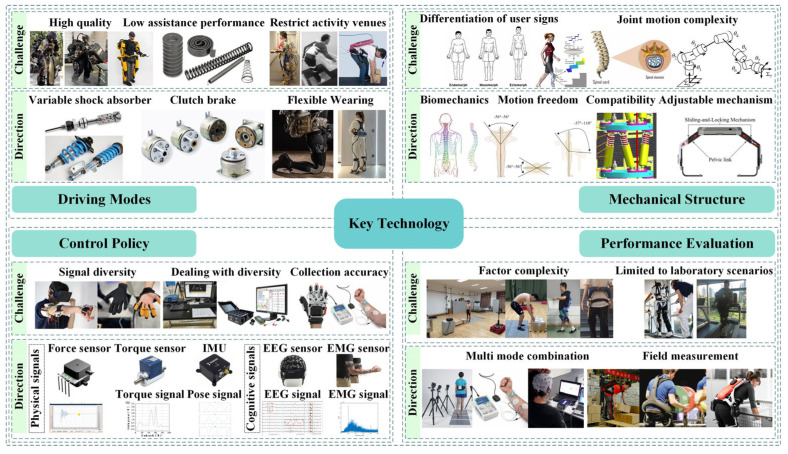
Key technical block diagrams.

In the research of lumbar spine assisted exoskeleton, human–machine interaction is particularly important, and four key technologies need to be used, which are a lightweight power mode, mechanical structure suitable for wearers, and flexible human–machine coordinated control strategy, as well as effective performance evaluation methods, as shown in Figure 12.

### 4.1. Driving Modes

Currently, the driving modes applied in the waist-assistive exoskeleton mainly include electric motor drive, hydraulic drive, pneumatic drive, and artificial muscle drive. Safety has always been a major concern in human–machine interaction, which is directly related to driving technology [46].

Due to its simple structure, fast response, and high efficiency, electric motor drive systems can provide significant assistive power for exoskeletons. Currently, many powered waist-assistive exoskeletons adopt electric motor drives, usually combining the electric motor with a reduction gear to achieve the necessary force/torque. Cray X [14], H-WEXv1 [47], and SIAT-WEX [43] all use electric motors and harmonic reduction gears to provide power. Harmonic drive reduction gears can achieve compact designs but are costly. Reduction motors may lead to undesirable forces to the wearer due to friction and inertia. Consequently, many researchers and scholars have proposed devices that combine electric motors with elastic elements, such as series elastic actuators (SEAs) and parallel elastic actuators (PEAs).

Masood et al. [48] designed an innovative parallel elastic actuator (PEA) using elastic cords made of natural rubber, which can store and release energy during ascent. Toxiri et al. [49] matched the multifunctionality of motors with the energy of springs as torque sources, for robot devices related to target tasks. H-WEXv2 [47] and lower-back exoskeletons [22] use series elastic actuators (SEAs) in their designs, incorporating clutches to assist when users need it.

Cable-driven actuators (CDAs) are emerging electric actuators, with their cables twisted by motors, hence serving as a means of converting rotation to linear transmission [25]. Yao et al. [50] developed a novel powered suit prototype, utilizing two twisted cable-driven actuators (CDAs) to assist torques at the hip and waist joints. Seong et al. [25] designed a humanoid assistive mechanism. CDAs are lightweight, are mechanically simple, and have high transmission ratios and low inertia but have a short lifecycle. However, all compliant components of the exoskeleton need to be pre-tensioned for CDAs to function as intended.

The pneumatic drive is mainly used in waist exoskeletons, combined with transmission mechanisms. The pneumatic drive offers safety, flexibility, lightweight, high output force, and good human–machine interaction safety. However, it typically requires an external air source, limiting the wearer’s range of motion. Moreover, pneumatic drive struggles with the precise control of the exoskeleton’s speed and position, and its assistive efficiency is lower. Pneumatic artificial muscles often use supportive materials that restrict deformation as the skeleton, with expandable airbags (or similar structures) inside, executes various compliant motions through the expansion and contraction of these airbags. This structure inherits the basic advantages of pneumatic components while also possessing simplicity, high flexibility, and good biomimetic characteristics that other mechanical actuators cannot match, making it one of the most widely used flexible driving modes [23], with typical structures including the McKibben type [51], linear fiber type [16], and balloon type [52].

Hydraulic drive ensures smooth transmission, compact structure, low inertia, and ease of control. However, hydraulic transmission is sensitive to changes in hydraulic oil temperature, and it may experience oil leakage, environmental pollution, and the inability to ensure transmission ratios, which may lead to low efficiency. Hydraulic drive is mostly used in exoskeleton robots for purposes such as seismic relief and soldier operations, where they bear significant loads.

Passive exoskeletons typically use elastic elements for assistance; elastic belts, springs, and elastic beams are most used. PLAD [26] combines rubber bands with rigid support structures to alleviate back muscle strain. The Auxivo Lift Suit [8] uses two textile springs parallel to the back muscles to provide back support. SSL [27] uses the elastic force of tension bands to provide energy for the back. Belt-driven systems are small in weight and economical but have limited assistance efficiency.

Springs being used as the power source for passive exoskeletons is also widespread. A wearable lightweight unpowered waist assist device (LWA) [53] applies torsion springs at the hip joints to apply torque during lifting. Leavo [54] combines air springs with cams at the hip joints to transmit torque, while Back X [30] also uses air springs to provide hip torque. VT-Lowe [38] uses composite plate springs made of carbon fiber as the only energy storage component in the exoskeleton. Flexible rods extend from the back to the legs, and flexible beams allow the body to move freely during bending and twisting [37]. Spexor [55] has a spinal structure composed of a ball joint and a linear slider combined with elastic beams, providing flexibility and compensating for potential misalignment.

In summary, most powered exoskeletons currently use electric, pneumatic, hydraulic, and other drivers, all of which are limited by their mass and volume, as shown in Table 2. Technical challenges still exist regarding the lifespan and performance of pneumatic artificial muscles during high-speed movements. Another significant aspect is the issue of energy supply for supporting drivers. Pneumatic exoskeletons usually require an external air source, which limits the wearer’s range of motion. The larger the load and the longer the usage time are, the greater the energy required is and the larger the mass of the electric motor is, which brings a challenge to the lightweight design of powered exoskeletons. Compared to belt-driven and elastic beam-type passive waist-assistive exoskeletons, those with larger assistive arms can achieve better assistance effects, albeit with relatively expensive materials. Elastic beam structures are lightweight, compact, and relatively simple. However, excessive loads may cause beam fracture, with stress peaks occurring at the bottom of the beam; hence, special attention must be paid to bottom fixation [56].

### 4.2. Mechanical Structure

The design of the mechanical structure must meet the characteristics of being lightweight yet sturdy, comfortable yet stable, and convenient yet safe. Waist-assistive exoskeletons driven by electric motors tend to have a larger mass compared to those driven by other means due to the presence of components, such as motors, transmission mechanisms, and control devices. Therefore, innovative design of structures and mechanisms, as well as the selection of new materials, are necessary to achieve lightweight waist-assistive exoskeletons.

#### 4.2.1. Actuation Transmission Mechanisms

**Figure 13:**
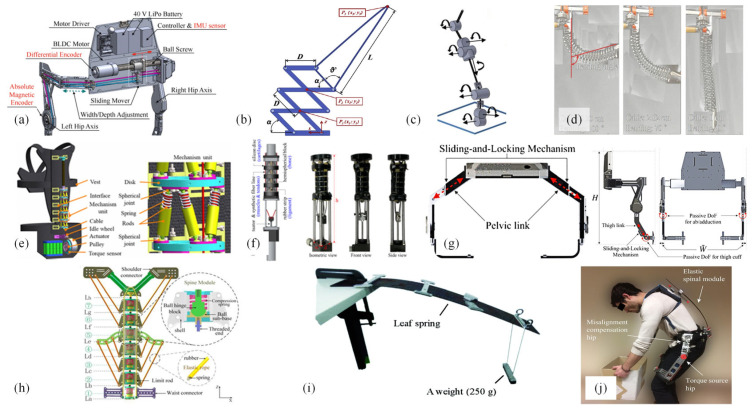
Mechanical structure design. (**a**) Linear transmission of ball screws [47]. (**b**) Scissor hinge mechanism [58]. (**c**) Viscoelastic couplings [59]. (**d**) Continuous soft exoskeletons [60]. (**e**) Ultra-redundant hybrid mechanisms [61]. (**f**) SJLM [62]. (**g**) Pelvic and thigh sliding adjustment mechanism [47]. (**h**) Elastic ball hinge limit [64]. (**i**) Spring steel plate support [52]. (**j**) Spexor [56].

Actuation transmission mechanisms can be classified into rigid and flexible structures, which are based on the nature of the actuator, and are typically designed in conjunction with the driving mechanism. Electric motor-driven systems commonly utilize rigid actuation structures and transmission components, offering high power transmission efficiency and minimal susceptibility to external influences. However, such choices often result in issues such as increased mass and structural complexity in electrically powered lumbar exoskeletons. Addressing these concerns, Hyun et al. [47] developed an improved electrically driven lumbar exoskeleton robot, the Hyundai Waist Assistive Exoskeleton version II (H-WEXv2, Figure 13a) featuring a linear drive mechanism composed of ball screw actuators and series elastic actuators capable of bidirectional lumbar movement (flexion/extension), thereby enhancing maneuverability. In the Japet. W exoskeleton, two belts located on both sides of the body are connected by two sets of actuators. These four actuators serve a dual purpose: applying traction while maintaining exoskeleton mobility. Series elastic actuators (SEAs) provide mechanical damping and power to the movement, with each actuator connected to the belt via a spherical joint, adapting automatically to different heights and allowing movement to follow the torso.

Schwartz et al. [57] evaluated the effectiveness of flexible support exoskeletons versus rigid back support exoskeletons in lifting tasks and found that the inclination of the torso in flexible exoskeletons is not only related to hip flexion but also to spinal flexion. In contrast, the support provided by rigid exoskeletons is activated through hip flexion rather than directly through torso inclination, since rigid structures cannot be directly pulled tight by spinal flexion. Furthermore, using rigid structures may even restrict spinal flexion. To address these issues, Roveda et al. [58] proposed a design approach for an active back-support exoskeleton based on spine-inspired kinematics, utilizing a scissor-like hinge mechanism (Figure 13b), and investigated a single-degree-of-freedom device. The mechanism’s design references the spatial displacement of human motion to ensure that the device follows its motion during task execution, albeit with the spine restricted to a single degree of freedom rotation in the sagittal plane, limiting the motion range of the human torso and lacking conformation to the waist.

Numerous scholars have analyzed the characteristics of the human spine to design transmissions that conform to the wearer’s back. Johnson et al. [59] developed an articulated spine exoskeleton prototype (Figure 13c), whose mechanical aspects (compression, resistance, range of motion) can be quickly and easily adjusted. The main components of the column include variable segment axial (sagittal, lateral, longitudinal) resistance couplers and variable angle couplers. Flexural couplings integrated into coupling components allow for the selection of angles between adjacent segments. A viscoelastic coupler was developed to allow independent control of resistance and range of motion for each joint while avoiding the vibrational effects typically exerted along the spine by standard elastic springs. Yang et al. [60] proposed a continuous spine-inspired soft exoskeleton (Figure 13d) for assisting in bending and lifting, which is a continuously bending hyper-redundant continuum mechanism adapted to the human spine. In 2022, Yang et al. [61] introduced a novel back exoskeleton, designing a hyper-redundant hybrid mechanism as the exoskeleton mechanism (Figure 13e), compatible with the multi-degree-of-freedom anatomical structure of the human spine. Jung-Yeong et al. [62] proposed a spine-like joint connection mechanism (SJLM) (Figure 13f), connecting multiple spherical blocks with synthetic fiber lines and rubber strips to mimic the function of the spine’s vertebrae, tendons, and ligaments.

Several of the aforementioned exoskeletons adopt continuous redundant mechanisms inspired by the spine. However, due to the complex kinematic characteristics and the need for parts to be machined according to the actual dimensions of the wearer, exoskeletons are difficult to adapt to different users. An adaptive transmission mechanism that accommodates the range of motion in all directions while effectively transmitting force remains a challenge for the future.

#### 4.2.2. Limitation Structures

To accommodate different workers, lumbar assistive exoskeletons should possess universality. Many researchers have optimized the design of exoskeletons for adjustability in mechanical structure, with limitation structures being one aspect. These structures serve to prevent sliding between the exoskeleton and the body, which are mainly placed near active joints. Without impeding the movement of major joints, they confine the assistive components to the joints requiring assistance. The thigh, waist, and thoracic spine are commonly chosen locations for setting limitation structures. The H-WEXv2 exoskeleton designed by Hyun et al. [47] incorporates two limitation locking structures (Figure 13g). A locking hole is set at the thigh junction to enable the sliding of the thigh link up and down, while a sliding locking structure at the pelvis facilitates inward, and outward sliding to accommodate users of different body sizes. Another purpose is to restrict the range of motion. In testing Leavo, the literature [63] found discomfort in the chest area because the exoskeleton joint has an end-limiting stopper. Further bending causes deformation in the flexible beam, resulting in a sharp increase in force on the chest pad after the joint bends approximately 140 degrees. Limitation in structure design not only protects the body’s safety but also helps maintain the body in a standard posture to reduce damage caused by poor posture. As previously mentioned, the issue with existing exoskeletons mainly using high stiffness mechanisms restricts the natural movement of the body. Inspired by the biological spine, many researchers have designed flexible spine-like mechanisms. Song et al. [64] designed a biomimetic spine exoskeleton consisting of multiple elastic spherical hinge units. Seven tapered units are connected end to end, each with four degrees of freedom, and connected by limitation rods (Figure 13h), restricting their range of motion to meet the body’s requirements for forward and backward bending, lateral bending, and back flexion and extension.

#### 4.2.3. Supporting Structures

In general, for lumbar support exoskeletons, complete support is not necessary. Providing full load support implies high stiffness in the structure, which can cause discomfort to the wearer [65]. Another concern is that excessive support may lead to muscle degradation. Therefore, existing exoskeletons are typically designed to provide up to 30% support [57].

During lifting tasks, the body is not continuously exerting force. Considering the walking gait during transportation, a normal gait cycle consists of a stance phase and a swing phase. Assistive joints only need to assist in the swing phase, and support during the stance phase to help the body save energy. This not only enhances assistive efficiency but also avoids generating resistance against the body, thus improving safety. The same principle applies to the lifting phase of human tasks. According to research [66], when lifting heavy objects, a large torque is required for lifting, but a small torque is needed for lowering. This requires control strategies to adjust torque output. In mechanical structure design, most exoskeletons use the backplate as auxiliary support. The Cray X exoskeleton [14], for example, provides additional mechanical support by extending the frame above the hips. Additionally, the backplate is typically configured as a component connecting the lower limbs and the shoulder straps of the upper limbs. Because steel plates can cause discomfort due to restrictions and compression on the body, components with rigid–flex coupling have become a research focus in recent years. The AB WEAR III exoskeleton [52] employs spring steel plates (Figure 13i) to provide limiting and supporting functions, counteracting shear forces on the lumbar spine. The Spexor exoskeleton [56] (Figure 13j) uses a passive, parallel elastic torque source to provide support at the hips.

**Figure 14:**
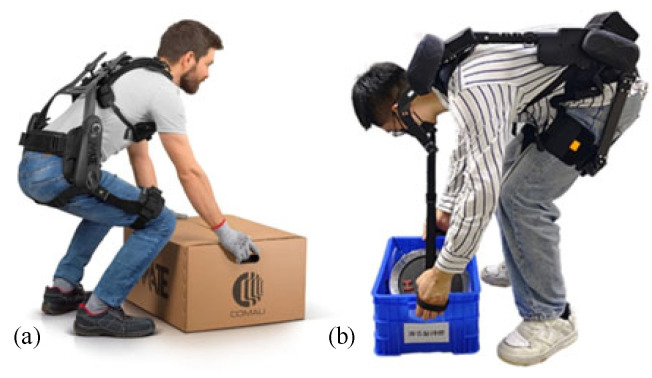
Case studies of practical application scenarios. (**a**) MATE-XB and (**b**) BEX-01.

Regarding case studies on practical application scenarios in logistics, Comau’s non-powered wearable exoskeleton (MATE-XB) features a modular design that provides back support through an intelligent torque assistance system for industrial repetitive handling. Its advantages include an ergonomic lightweight construction (only 7.5 kg) and a fast wear design, but there are significant disadvantages: there is only passive elastic assistance (no active drive), the strength of the assistance is not adjustable, and the lack of intention recognition sensors may limit its flexibility in complex work scenarios, as shown in Figure 14a. The waist power exoskeleton (BEX-01) of kenqing Technology Company adopts gas-electric hybrid drive and intelligent torque distribution technology, realizes motion intention recognition through multi-sensor fusion, provides 30 kg of power and reduces waist load by 40%, and is suitable for logistics handling and other scenarios. The disadvantage is that the 100 ms response delay may affect dynamic coordination, the 8 h endurance is slightly insufficient for high-intensity operations, and the adaptability data to extreme environments is not clear, as shown in Figure 14b.

In summary, the design of exoskeletons mainly involves the connection methods of various types of exoskeletons and the compatibility design of mechanisms. Compared to the rigid support structures of traditional exoskeletons, an increasing number of researchers have designed flexible skeletal exoskeletons based on ergonomics. Adjustable mechanisms adaptable to personalized physical characteristics have emerged to address the issue of non-adjustable heavy exoskeleton structures. In contrast to traditional mechanical joints, more lumbar assistive exoskeletons adopt biomimetic spine joint mechanism designs.

### 4.3. Control Policy

Passive exoskeletons typically rely on manual switching to change assistive modes. While manual switching is stable, intuitive, and easy to use, it can result in intermittent task execution and reduced actuator performance. Moreover, during heavy lifting tasks, the operation of manual switches may not be feasible, posing safety concerns for the wearer [67]. In contrast to passive devices, powered lumbar exoskeletons can simultaneously implement multiple assistive strategies, making them more versatile and effective. For example, Wang et al. [68] designed a wearable upper limb exoskeleton (ULE) robot that uses flexible cables to transmit auxiliary torque. Based on the K-nearest neighbor (KNN) algorithm and integrated fuzzy PID control strategy, the ULE recognizes carrying attitude and automatically provides accurate active auxiliary force. The control strategies for powered lumbar assistive exoskeletons mainly include human intent recognition control strategies and human–machine collaborative control strategies.

#### 4.3.1. Perception of Human Motion Intent

Human motion intention perception technology mainly involves two control strategies: direct control and indirect control. The direct control strategy involves acquiring data signals from the wearer, with bioelectric signals and biomechanical signals being the most widely used. Indirect control typically involves measuring interaction forces between the wearer and the exoskeleton, joint angles, and other external environmental factors [69].

(1) Indirect Control

Indirect control can be categorized into static measurement and dynamic measurement based on the nature of the measured parameters. Static measurement, commonly employed in most exoskeletons, usually utilizes inertial measurement units (IMUs) or encoders integrated into exoskeleton joints to measure motion information of body parts such as the trunk and joint angles. For example, systems like WSAD [15] and WPAD [70] detect when users maintain static postures and activate accordingly to assist. Exoskeletons like HAL [71], Cray X [14], and H-WEX [72] compensate for gravitational forces acting on the trunk by measuring joint angles. However, these methods cannot distinguish whether the user requires assistance in lifting heavy objects, indicating that direct control may have advantages in identifying user intentions.

(2) Direct Control

Direct control involves using surface electromyography (EMG) to capture user intentions. Currently, EMG control for lumbar assistive exoskeletons mainly includes measuring the activity of the spinal muscles and forearm muscles, which are the primary force-generating muscles during lifting. For instance, HAL [73] measures the activity of spinal muscles and proportionally determines the assistive torque to provide. Robo-Mate [48] detects the activity of forearm muscles and sends commands to increase assistive torque. Lazzaroni et al. [74] designed a control strategy specifically for assistive pulling, utilizing forearm muscle activity to adjust the assistive torque. These torques are expected to adapt to the user’s assistance needs and the mass of the pulled object, as forearm muscle activity is considered an indicator of grip force. Notably, control strategies based on spinal muscle activity cannot perceive the weight of objects, while those based on forearm muscle activity may potentially achieve assistance magnitude control based on the weight of objects in the future.

Due to technical complexities, particularly signal variability and electrode burden, the professional use of surface electromyography-based control strategies in industrial settings presents challenges [75]. Therefore, mixed strategies for user intention recognition are being increasingly applied. For example, Toxiri et al. [76] explored a mixed control strategy for back-support exoskeletons during lifting tasks, combining indirect and direct control. They found that adopting a mixed control strategy based on indirect control of trunk posture and detecting additional effort required for lifting and moving external objects through forearm EMG activity and fingertip pressure makes assistance more effective.

#### 4.3.2. Human–Machine Collaborative Control Strategies

Broadly speaking, the core of exoskeleton control lies in perceiving human motion intentions and utilizing this perception to control the exoskeleton’s movements. In recent years, researchers have proposed many control schemes for wearers’ task switching, such as Artificial Neural Networks (ANNs) [77] and rule-based algorithms combined with Quadratic Discriminant Analysis [78]. These schemes involve acquiring motion data from the wearer without the exoskeleton, simulating the acquired data in a training machine to reduce the delay of measurement methods like posture sensors, fundamentally analyzing the phases of human tasks and training models. When the wearer dons the exoskeleton, the need for assistance is determined based on phase trajectory segmentation.

Researchers of the Japanese HAL exoskeleton robot have proposed two intention recognition methods. The first method is based on back angle and leg muscle EMG signals to determine lifting tasks [79]. Using sensors for trunk bending and thigh muscle EMG data, when the interaction system detects muscle signals within the working range, it triggers an assistance mode. The second method is an ANN-based approach utilizing EMG signals near the hip and knee joints. Collected EMG data are input into the ANN for training, determining the wearer’s lifting mode. A research team from the Italian Institute of Technology proposed a Support Vector Machine (SVM) classification method based on hip joint angle, trunk angle, and forearm EMG signals [80]. The SVM was trained using collected data to classify the wearer’s lifting mode. They also proposed an SVM classification method based on knee and trunk motion data [81], designing an information acquisition system consisting of back and four-leg IMUs. Motion data of the knees and trunk were input into the SVM for training, classifying the human lifting mode. Another group from the Italian Institute of Technology [78] proposed a Finite State Machine classification method based on embedded exoskeleton sensor information, setting thresholds based on different motion actions to determine the lifting mode. In contrast, for semi-passive exoskeletons, the key to control strategies lies in algorithms predicting user motion and maximizing the use of passive viscoelastic elements. In 2020, Jamšek et al. [40] developed a new control scheme consisting of a Gaussian Mixture Model (GMM) combined with a state machine controller.

Another solution for switching between different tasks is to develop strategies for automatically identifying wearer activities. Strategies for automatically identifying wearer activities mainly include lifting motion recognition control strategies and walking transportation motion recognition control strategies. Poliero et al. [82] evaluated multifunctional and non-multifunctional control strategies of exoskeletons and found that non-generic control strategies hinder the wearer’s natural gait during walking. This suggests that selecting different strategies according to the tasks performed is necessary [40]. Additionally, even for the same task, such as lifting, different assistance magnitudes are required in different stages. A single assistance mode may hinder the human body at certain stages, making mixed control strategies increasingly popular.

Chen et al. [78] addressed the issue that most existing active exoskeletons for lifting assistance cannot automatically detect user lifting actions and proposed a lifting detection strategy using only integrated sensors in the exoskeleton. Chen et al. [83] designed a real-time two-step algorithm to classify different lifting techniques, using only sensors embedded in the exoskeleton, which can detect the start of lifting motion and classify techniques used for lifting. However, both of these control methods exhibit delay during the process from signal collection to activation. Lazzaroni et al. [75] proposed using raw signals from accelerometers integrated into IMU sensors, which simultaneously measure linear acceleration and gravity, requiring no processing, thereby improving latency. Nevertheless, throughout the entire gait cycle of walking transportation, hip-assisted exoskeleton robots will assist the wearer’s walking transportation, which reduces the exoskeleton’s endurance. To address this issue, Wei et al. [66] proposed a power control method, dividing the control strategy into two parts. Firstly, the real-time tracking of interaction torque signals is achieved using the closed-loop feedback control of motor output torque, maintaining zero interaction force. In another control strategy, the required output torque is mainly determined based on the actual system joint rotation angular velocity and hip joint output power during semi-squat lifting. In addition, Arefeen et al. [84] proposed a multi-joint dynamic exoskeleton control strategy through human–machine coupling simulation. Taking the weightlifting task as an example, inverse dynamic optimization was used to predict the optimal auxiliary torque of human-exoskeleton symmetric lifting motion and multiple joints.

**Figure 15:**
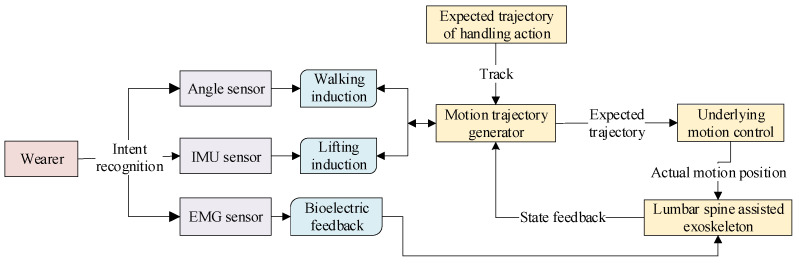
A block diagram of the exoskeleton control system.

In summary, current power-assisted lumbar exoskeletons mainly have two control methods: torque control and position control. However, most exoskeletons can only provide constant torque and fixed position reference curves, unable to adapt to different users. Figure 15 shows the overall control policy deployment. Adaptive controllers and control methods have emerged to address these issues.

For repetitive joint movements, encoding and reproducing trajectories is the most commonly used and convenient method. Among them, Dynamic Movement Primitives (DMPs) are one of the methods for trajectory parameter representation [85]. Typically, the phase and frequency of trajectories are extracted and controlled by Adaptive Frequency Oscillators [86]. Ronsse et al. [87] used adaptive oscillators to adjust the frequency of position trajectory learned according to the subject’s motion. This method estimates the assistive joint torque based on the difference between the current position and the predicted future position phase shift. However, most iterative learning control algorithms require switching between assistive and learning modes and exhibit some degree of delay. Peternel et al. [88] proposed an exoskeleton control method for adaptively learning assistive joint torque curves in periodic tasks. Using human muscle activity as feedback, the assistive joint torque behavior is adjusted to minimize muscle activity, without the need for biomechanical or dynamic models. Lanotte et al. [89] proposed and validated a new impedance control framework based on Adaptive DMPs (aDMPs) for assisting discrete movements. This dynamically updates the predicted motion trajectory and enables the adaptive torque controller to provide functionally optimized assistance. In 2023, Li et al. [90] proposed a new method for generating lifting reference curves, which can generate multiple posture-mixed lifting task assistance curves for each user, such as squatting, bending, and left–right asymmetry, and designed an adaptive predictive controller that can track reference curves under different loads for different users.

In conclusion, the control strategies for automatically identifying wearer motion intentions face issues such as motion interference and parameter complexity. Integrated sensors, training models, and other control strategies exhibit delay issues during control. How to provide precise and rapid assistance to wearers in different stages of motion remains an unresolved issue. Traditional exoskeleton control strategies mostly involve static control methods that do not consider environmental feedback, complex multi-parameter control methods, control methods without safety measures, increasingly improved methods based on interactive control methods considering wearer feedback, simplified control methods to alleviate worker pressure, and control methods supporting warnings.

The existing waist-assisted transport exoskeleton mainly relies on traditional sensors (such as IMU, sEMG) and shallow machine learning (such as SVM) in terms of motion intention perception, which is not adaptable to complex dynamic tasks and prone to recognition delay or misjudgment. At the same time, the control strategy mostly adopts rigid power assist methods such as rules or PID, which lacks dynamic adjustment ability to individual differences and real-time load changes, resulting in poor human–machine cooperation and user fatigue. In order to break through these limitations, the future needs to combine deep learning (such as LSTM and Transformer) to improve the accuracy and dynamic adaptability of intention perception [91] and use reinforcement learning (RL) and imitation learning to optimize adaptive control strategies [92], further enhance personalized adaptation capabilities through digital twin and transfer learning, and finally achieve efficient and flexible human–machine collaboration [93].

### 4.4. Performance Evaluation

The performance evaluation of exoskeletons involves numerous indicators and methods. Pesenti et al. [94] categorized the evaluation methods of exoskeleton performance into five standard domains: functionality, force or torque, metabolism, muscle activity, and subjective assessment.

In the domain of functionality, indicators related to tasks are measured, such as kinematic measurements, task completion time, posture maintenance duration, repetition count, walking distance (with or without load), etc. Kinematic measurements are typically conducted through motion capture to estimate potential posture changes introduced by the exoskeleton.

The force/torque domain primarily calculates the force/torque changes at the major joints involved in tasks. For assessing assistive performance in lumbar assistive exoskeletons, measurements mainly focus on the compressive force at the L5-S1 lumbar joint or the flexion–extension torque around this joint, joint moment, mechanical joint work, and ground reaction forces. Ulrey et al. [95] evaluated the assistive performance of BNDR by measuring changes in compressive and shear forces at the L5-S1 level. Madinei et al. [39] assessed the assistive performance of exoskeletons by comparing peak compressive and shear forces before and after wearing. Marinou et al. [96] evaluated the effectiveness of exoskeletons in reducing risk by comparing peak lumbar loads and maximum lumbar flexion angles. Nabeshima et al. designed a standardized performance testing protocol for WRLS [81]. In the test, the experimental apparatus follows trunk movements during artificial lifting, holding, and raising processes. Subsequently, two performance indicators, the Assistance Torque Index (ATI) and Lumbar Compression Reduction (LCR), are evaluated.

Metabolic cost or rate is typically assessed by comparing the metabolic rates of subjects with and without wearing exoskeletons to evaluate their effectiveness. Baltrusch et al. [36] evaluated the impact of SPEXOR devices on repetitive lifting metabolic costs by measuring breath volume, oxygen consumption, and carbon dioxide production.

Muscle analysis using electromyography (EMG) is the preferred measurement method in lumbar assistive exoskeleton evaluation studies. In recent years, many studies have used changes in the activation of major muscles during a series of repetitive lifting tasks with and without exoskeletons as an evaluation criterion for exoskeleton performance [53,82,97,98,99]. Common methods also include testing the wearer’s heart rate [100], body temperature, blood pressure, endurance, oxygen consumption [101], and other physiological signal changes [102]. However, these evaluation methods are often used in combination to enhance the accuracy and persuasiveness of the test results. For example, several studies evaluating the effectiveness of exoskeleton use include muscle activity and net joint torque reduction during controlled conditions such as manual precision, static holding, and symmetrical MMH tasks [56,100,103]. Maja et al. [104] conducted four types of measurements in the performance evaluation of the HWA exoskeleton: electromyography (erector spinae, rectus abdominis, and oblique muscles), trunk kinematics, self-reported ratings, and heart rate. Luger et al. [99] studied the impact of passive back-support exoskeletons (Laevo V2.56) on muscle activity, posture, heart rate, performance, usability, and wearer comfort. Muramatsu et al. [105] assessed muscle usage through electromyography [23] and evaluated muscle fatigue through near-infrared spectroscopy and dynamic length of body sway (DLNG). Han et al. [106] evaluated exoskeleton performance by testing the resultant and effective moments on the lumbar spine, while also measuring the wearer’s breathing rate and heart rate to assess muscle fatigue.

**Figure 16:**
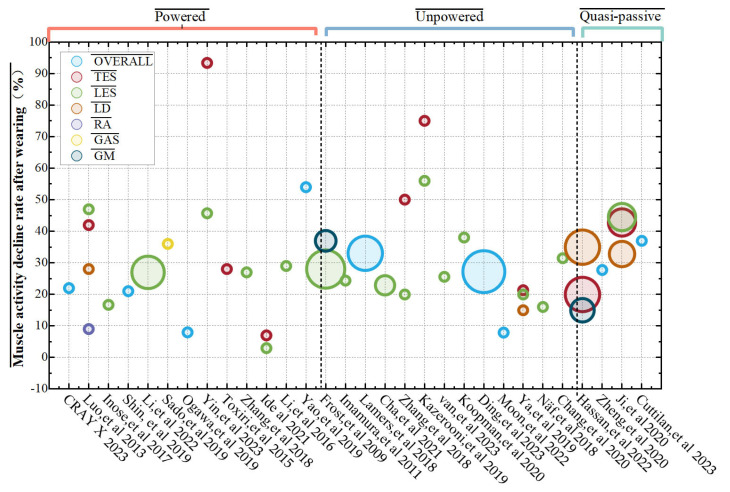
Statistical graph of muscle activity decline rate after wearing exoskeletons in the different literature [9,14,15,16,17,19,21,22,23,25,26,27,28,29,30,31,32,33,35,36,50,53,70,100,108,109,110,111,112,113].

Subjective assessment refers to the measurement of perceived task difficulty, system usability and acceptability, perception of fatigue, and discomfort. Subjective assessment methods involve having wearers fill out subjective assessment questionnaires. Several widely used subjective assessment methods include the Visual Analog Scale (VAS), Borg Rating of Perceived Exertion (RPE) scale, Likert scale, and overall perceived exertion (RPE). Baltrusch et al. [55] used the VAS to evaluate the user satisfaction of workers with and without a history of lower back pain (LBP) when wearing the SPEXOR passive trunk exoskeleton. Moulart et al. [107] used the VAS visual analog scale to assess the impact of lumbar exoskeletons on the perception of low back pain in real work situations. Walter et al. [14] analyzed the effect of the Cray X on RPE during lifting. Subjective assessment methods are often based on the wearer’s subjective feelings and are usually not used as standalone performance testing methods but as auxiliary methods to assess their comfort. In the reviewed literature, 14 evaluation criteria and indicators were identified with muscle and functional domains, which were the most common areas, as shown in Table 3, and muscle domain evaluations are illustrated in Figure 16.

The above five evaluation domains require extensive data collection and processing, leading to significant time costs. To address these issues, many scholars have proposed testing models to evaluate the impact of exoskeletons on the human body after wearing. Moya-Esteban et al. [114] proposed a real-time electromyography (EMG)-driven musculoskeletal model, which can accurately estimate lumbo-sacral joint torque and reasonable compression force. It can estimate lumbo-sacral joint torque and compression force in real-time based on in vivo experimental data and detect the biomechanical effects of passive back support exoskeletons. Alemi et al. [115] proposed a new metabolic cost model for repetitive lifting and the influence of wearing a passive back support exoskeleton on it. Zelik et al. [116] introduced Exo-LiFFT, a human factor assessment tool that can evaluate or predict the effects of exoskeletons on low back disorder (LBD) risk without electromyography testing. Tröster et al. [117] conducted biomechanical analysis of bending and squatting using a generic back-support exoskeleton model. Xiang et al. [118] investigated an exoskeleton evaluation method based on functional analysis of variance, using FANOVA (functional analysis of variance) to evaluate the effectiveness of exoskeletons and estimate biomechanical variables by importing motion data into an exoskeleton-human body model. Sposito et al. [119] introduced a model-based method to evaluate the kinematics of exoskeletons by representing activity constraints as disturbances and unwanted forces at anchor points. The emergence of these evaluation tools has accelerated the performance and functional evaluation of exoskeletons, as shown in Table 4.

In summary, there are many methods for evaluating exoskeleton performance. However, these methods are typically conducted in laboratory settings, while the scenarios in which workers use exoskeletons are more complex. Laboratory environments may not simulate the unexpected situations that workers encounter in actual complex scenarios. In future evaluations, it is important to simulate these real usage scenarios as much as possible.

This study analyzes the driving mode, mechanical structure, control strategy, and performance evaluation of lumbar assisted transport exoskeletons, which still face many challenges. In terms of drive, the existing system generally has problems such as large mass, low auxiliary efficiency, and limited motion range. In the future, lightweight and efficient drive schemes such as variable damping mechanism and intelligent clutch can be explored. The limitations of the mechanical structure are reflected in the complex joint movement and poor adaptability. In the future, the adjustable mechanism can be combined with the biomechanical optimization design to improve the versatility and man-machine matching degree. In terms of control strategy, the existing system relies on multi-platform signal processing, resulting in high delay and insufficient coordination. In the future, real-time intention recognition and adaptive control can be realized based on multi-mode sensing (such as sEMG, IMU, and force feedback) and machine learning. In addition, the performance evaluation is still limited to the laboratory environment, and the measurement indicators are complex and insufficient in practicality. In the future, it is necessary to integrate the test standards into the design stage and combine digital twin and other technologies to achieve a dynamic and scenario-based evaluation system. In short, through the combination of lightweight drive, intelligent structure optimization, multi-modal collaborative control, and a standardized evaluation system, the exoskeleton can be promoted to the direction of efficient, adaptive, and practical development.

## 5. Conclusions

This paper discusses the characteristics of the lumbar spine joints and the kinematic and dynamic features of lifting movements. It demonstrates that lumbar assistive exoskeletons can significantly alter the biomechanics of lumbar spine joints during daily activities through various mechanisms, effectively preventing LBP and helping workers engaged in MMH tasks. By examining the limitations, development suggestions, and future trends from the perspectives of drive systems, mechanical structures, control strategies, and human–machine interaction, this paper lays the theoretical foundation for further development of lumbar assistive exoskeletons. It is significant for enhancing the application value of lumbar assistive exoskeletons.

(1) In terms of drive systems, considering that the target users are often workers engaged in prolonged bending activities, it is crucial to minimize the fatigue induced by the exoskeleton on the wearer’s body. Therefore, the design of the drive system should prioritize characteristics such as light weight, compact size, and high efficiency. Unpowered exoskeletons rely solely on flexible/elastic structures to conform more closely to the wearer, but they also have limitations. Powered lumbar assistive exoskeletons, along with their corresponding assistive strategies, can offer more functionality, but their motors and transmission mechanisms tend to be bulky, leading to fatigue when used for extended periods in industrial settings. Hence, the design of exoskeleton drive systems should integrate the advantages of various drive modes. For example, leveraging parallel elastic actuators can reduce the size and weight of the device while meeting the required output torque.
(2) In terms of mechanical structure design, the goal is to efficiently transmit assistance while minimizing unnecessary constraints and user discomfort. Existing exoskeletons primarily focus on sagittal plane movements, which, to some extent, restrict human activities. Therefore, enabling the unrestricted movement for the wearer is a primary concern. The choice of transmission structure depends on the driving mode. Currently, most electrically driven exoskeletons utilize rigid structures to transmit assistance, while unpowered exoskeletons mostly employ flexible structures for assistance, albeit with limited effectiveness. Hence, the design of exoskeleton mechanical structures can incorporate a combination of rigidity and flexibility. For example, exoskeletons such as Spexor [56] and Leavo [100] use rigid structures as the supporting part, with motors incorporating elastic components to transmit torque and absorb human energy for assistance, thereby expanding the range of human movement. Simultaneously, simplifying the power transmission mechanism can effectively reduce the weight of the exoskeleton [47].
(3) In terms of control strategies, the majority of existing lumbar exoskeletons primarily provide assistance for lifting tasks (bending and squatting), yet workers’ postures in industrial settings also involve various movements such as walking, pushing, pulling, lifting, and carrying [83]. The design of exoskeletons should ensure that they can automatically identify moments when assistance is needed during different tasks performed by the wearer, without interfering with natural human motion. Device parameters should be adjusted according to different users, and if necessary, the exoskeleton operation can be manually halted to ensure operational safety.
(4) In terms of human–machine interaction, firstly, regarding degrees of freedom, there should be sufficient freedom of movement to not constrain natural human motion. Secondly, concerning human–machine adaptation, efforts should maximize conformity to the articulation of human joints. Finally, lightweight design should consider the optimal selection of structure, size, and materials. Materials should prioritize lightness, breathability, flexibility, and high wearing comfort while meeting basic stiffness requirements. Novel smart materials have been introduced and successfully applied in existing exoskeletons [120]. However, currently, most exoskeletons have not considered the fatigue experienced by users during prolonged wear. Whether powered or unpowered, exoskeletons have a certain weight; after all, they are “external” skeletons and cannot truly integrate with the human body. The real-time intelligent monitoring of users’ fatigue levels while wearing exoskeletons may be a worthwhile direction for future development.

Subsequent technical improvements will focus on computational optimization and standardized testing. Firstly, lightweight edge computing architecture (such as TinyML) should be developed to reduce power consumption while ensuring control accuracy. Secondly, a dynamic evaluation protocol should be established that complies with ISO/CE standards, covering core metrics such as biomechanical efficacy (such as lumbar torque reduction rate), energy consumption ratio, and long-term wear safety. Finally, multi-center clinical trials (medical scenarios) and large-scale deployment in the logistics industry (industrial scenarios) should be performed to achieve technology transformation, focusing on solving industrial pain points such as personalized adaptation and system reliability.

In order to improve the practicability and reliability of lumbar assisted transport exoskeleton, it is necessary to break through the three technical bottlenecks of hardware integration, software standardization, and edge computing. In terms of hardware, multi-mode fusion sensor module and low-latency communication architecture should be developed to solve the problem of data synchronization. At the software level, a unified development platform should be established to realize the seamless connection from simulation to practical application. In the future, edge intelligent computing can be used to achieve local real-time decision-making through embedded AI. These improvements will significantly reduce system latency, reduce development costs, and improve device compatibility, clearing technical hurdles for clinical and industrial applications.

## Figures and Tables

**Table 1:** Joint range of motion.

Joint	Athletic Forms	Range of Motion (°)
Lumbar spine	Flexion/Extension	110~−37
	Left/Right Bend	−56~56
	Left/Right Rotation	−50~50
Hip joint	Flexion/Extension	130–140/10–30
	External/Internal Rotation	40–50/30–45
	Abduction/Adduction	45–60/20–30

**Table 2:** A comparison of the advantages and disadvantages of the lumbar spine-assisted exoskeleton drive modes.

**Drive Type**	**Advantages**	**Disadvantages**
**Motor-Driven**	(1) The energy transmission of cable connection is convenient and fast (2) It is easy to realize automatic control (3) Simple structure (4) No pollution	(1) Poor balance of movement (2) Large volume
**Hydraulically Driven**	(1) Large output power (2) No deceleration mechanism is required (3) Strong ability to withstand mechanical impact and damage	(1) Easy to leak oil (2) The hydraulic power source must be configured
**Pneumatic-Driven**	(1) Clean and pollution-free (2) It is easy to realize stepless speed change (3) Low cost (4) Simple structure	(1) Poor control accuracy and high noise (2) It is not suitable for working at low temperatures (3) Only suitable for low power transmission
**Pneumatic Artifical Muscles**	(1) Clean and pollution-free (2) Compared with the cylinder, it has a larger power volume and power-to-mass ratio (3) Simple structure	(1) Air is easy to compress and leak (2) Difficult to control precisely
**SEA-Driven**	(1) High control accuracy (2) High security	(1) The stiffness is limited by the elastic element (2) Complex structure
**PEA-Driven**	(1) Improve the reverse drive ability (2) Improve energy efficiency	(1) Lack of active control (2) Lack of instantaneous control
**TSA-Driven**	(1) Light weight (2) Mechanical simplicity (3) High gear ratio (4) Low inertia	(1) Short lifecycle (2) There are operating conditions

**Table 3:** Product function comparison of power exoskeleton and quasi-passive exoskeleton.

Name	Institution	Driving Mode	Structure	Control Policy	Quality	Peculiarity	Assisting Efficiency
MODLE-Y [13]	ATOUN Inc.	Electricity	Rigid	Motion	4.5 kg	Inverted Y Type	10 kg
CRAY X [14]	German Bionic Systems	Electrical	Rigid	Motion	9 kg	Additional Mechanical Support	25 kg
WSAD [15]	University of Science and Technology of China	Electrical	Flexible	Motion	-	Servo Motor, Tension Belt Drive	30%
AB-WAER II [16]	Chung-Ang University of Korea	Pneumatic	Rigid	External Control	2.9 kg	Greater Contraction Force	16.7%
Back booster Exoskeleton [17]	Korea Advanced Institute of Science and Technology	Pneumatic Artificial Muscles	Rigid	-	-	Gear and Belt Structural Design	21%
HipExo [18]	University of Moletuvo, Sri Lanka,	Electricity	Springs	Motion	-	Hybrid Actuators	30%
Robo-Mate [21]	Italian Engineering	Electrical	Rigid	Motion, EMG	11 kg	Forearm EMG Control	28%
HExo [41]	Sun Yat-sen University	Servo Motor	Rigid	Motion Recognition	-	Upper Limb And Lower Back Combination	36.92%
Powered lumbar exoskeleton [42]	Shenzhen Institute of Advanced Technology, Chinese Academy of Sciences	Motor,	Rigid	Motion Recognition	-	Mechanical Clutch	20 kg
SIAT-WEXv1 [43]		Flat Brushless Motor and Harmonic Drive Gear	Rigid/Flexible	IMU, Angle Sensor	5 kg	Ergonomic Mechanical Structure	44.5%

**Table 4:** Literature statistics of evaluation methods.

Evaluation Indicators/Times		Frequency/Times	Power Type/Times	Non-Powered Type/Times	Quasi-Passive/Times
Functional areas/5	Kinematics	4	2	2	/
	Static hold	1	/	/	1
Force or moment/18	Auxiliary force	9	6	1	2
	L5-S1 Bending–stretching Moment	7	2	3	2
	L5-S1 peak compressive force	2	/	1	1
Metabolism field/11	Metabolic costs	1	1	/	/
	Heart rate	6	3	1	2
	Respiratory rate	4	1	1	2
Muscle field/32	Muscle activity	1	/	/	1
	iEMG	31	14	12	5
Subjective evaluation/4	GRE	1	1	/	/
	Borg	2	2	/	/
	Perceive exertion	1	1	/	/

## Data Availability

The authors confirm that the data supporting the findings of this study are available within the article.
